# A novel missense mutation of the paired box 3 gene in a Turkish family with Waardenburg syndrome type 1

**Published:** 2013-01-29

**Authors:** Filiz Hazan, A.Taylan Ozturk, Hamit Adibelli, Nurettin Unal, Ajlan Tukun

**Affiliations:** 1 Department of Medical Genetics, Dr. Behçet Uz Children's Hospital, Izmir, Turkey; 2 Department of Ophthalmology, Dr. Behçet Uz Children's Hospital, Izmir, Turkey; 3 Department of Otolaryngology, Dr. Behçet Uz Children's Hospital, Izmir, Turkey; 4 Department of Pediatrics, Dr. Behçet Uz Children's Hospital, Izmir, Turkey; 5 Department of Medical Genetics, Faculty of Medicine, Ankara University, Ankara, Turkey; 6 Duzen Laboratory Groups, Genetics Division, Ankara, Turkey

## Abstract

**Purpose:**

Screening of mutations in the paired box 3 (*PAX3*) gene in three generations of a Turkish family with Waardenburg syndrome type 1 (WS1).

**Methods:**

WS1 was diagnosed in a 13-month-old girl according to the WS Consortium criteria. Detailed family history of the proband revealed eight affected members in three generations. Routine clinical and audiological examination and ophthalmologic evaluation were performed on eight affected and five healthy members of the study family. Dystopia canthorum was detected in all affected patients; however, a brilliant blue iris was present in five patients who also had mild retinal hypopigmentation. Genomic DNA was extracted from the peripheral blood of affected and unaffected individuals in the family as well as 50 unrelated healthy volunteers. All coding exons and adjacent intronic regions of *PAX3* were sequenced directly.

**Results:**

A novel missense heterozygous c.788T>G mutation was identified in eight patients. This nucleotide alteration was not found in unaffected members of the study family or in the 50 unrelated control subjects. The mutation causes V263G amino-acid substitution in the homeodomain of the PAX3 protein, which represents the 45^th^ residue of helix 3.

**Conclusions:**

We identified a novel missense c.788T>G mutation in *PAX3* in a family with Waardenburg syndrome with intrafamilial phenotypic heterogeneity.

## Introduction

Waardenburg syndrome (WS), which equally affects both sexes and all races, is an inherited disorder characterized by varying degrees of sensorineural deafness and pigmentary abnormalities affecting the skin, hair, and eye with an incidence of 1 in 40,000 [1,2]. Five major and five minor diagnostic criteria for WS were proposed by the Waardenburg Consortium [3]. Two major or one major and two minor criteria must be found in an individual to diagnose WS [3,4]. WS is classified into four major types depending on the presence or absence of dystopia canthorum and additional symptoms [1,5-8].

WS shows genetic heterogeneity. WS1 and WS3 are caused by mutations in the paired box 3 (*PAX3*) gene [9-12]. WS2 is due to mutations in the microphthalmia-associated transcription factor (*MITF*) gene [13-17] and the encoding snail homolog 2 (*SNAI2*) gene [18]. However, the molecular etiology of most patients with WS2 is still unknown [19]. WS4 is associated with mutations in the endothelin receptor type B (*EDNRB*) gene [17,20,21], the endothelin-3 (*EDN3*) gene [22-25], and the SRY (sex determining region Y)-box 10 (*SOX10*) gene [6,7,19,24]. Recently, deletions in *SOX10* were also identified in patients with WS2 and WS4 [26].

*PAX3*, located on the long arm of chromosome 2 (2q35), includes ten exons [27]. This gene is a member of the mammalian *PAX* gene family that encodes for DNA-binding transcription factors and plays a role in maintaining stem cell pluripotency, cell-lineage specification, proliferation, migration, apoptosis, and inhibition of terminal differentiation [28,29]. *PAX3* is expressed in neural crest cells including the spinal ganglia, the craniofacial mesectoderm, and the limb mesenchyme during embryogenesis and plays an important role in the migration and differentiation of melanocytes, which originate from the embryonic neural crest [17,28].

More than 70 pathogenic *PAX3* mutations including missense, nonsense, and frameshifts mutations, small insertions or deletions, and splice alterations have been reported in patients with WS1 [17]. Most *PAX3* mutations are located in exon 2; no mutations have been described in exons 9 and 10. Approximately 50% of the described mutations of *PAX3* are missense; the remaining are truncating variations [17]. Partial or total gene deletions have been reported in 10% of patients without identified point mutations [30-35]. The literature that studied patients with WS1 caused by mutations in *PAX3* was reviewed in 2009 by Pingault et al., and no relationship was found between the severity of disease and the type of mutation [17]. In this report, clinical features and intrafamilial heterogeneity of eight affected individuals in a Turkish family with WS1 with a novel mutation are presented.

## Methods

### Family description

**Figure 1:**
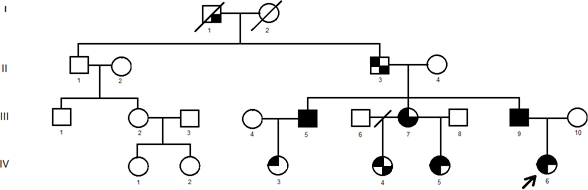
The pedigree of the family is shown. The squares indicate men, and the circles indicate women. Filled quadrants indicate phenotype associated with WS1. Upper left represents dystopia canthorum. Lower left represents brilliant blue iris. Upper right represents hearing loss. Lower right represents synophyris.

Eight affected including the proband, and 5 unaffected members of the study family, as well as 50 healthy volunteers were included in the present study. Informed consent conforming to the tenets of the Declaration of Helsinki, blood samples, and clinical evaluations were obtained from each study participant, under protocols approved by Dr. Behçet Uz Children's Hospital ethics committee (approval number and date: B-10-4-ISM-4-35-65-72; 29.03.2012/25). Informed consent conforming to the tenets of the Declaration of Helsinki, blood samples, and clinical evaluations were obtained from each study participant, under protocols approved by the Behçet Uz Children's Hospital ethics committee (approval number and date: B-10–4-ISM-4–35–65–72; 29.03.2012/25). The female patient was referred to us for genetic evaluation because of dysmorphic facial features at the age of 13 months. The proband and her family were evaluated at the Medical Genetics Clinic, Dr. Behçet Uz Children's Hospital, Izmir, Turkey. The proband (Patient IV:6) was diagnosed with WS1 according to the WS Consortium criteria [3]. Her family history revealed eight affected members in three generations (Figure 1). Routine clinical examination and detailed audiological and ophthalmologic evaluation were performed on eight affected and five healthy members of the study family. Two major or one major and two minor criteria must be found in an individual to diagnose WS. In four patients (IV:3, IV:4, IV:5, and IV:6), premature graying of the hair was not evaluated because of the patients’ youth.

Hearing loss was assessed with pure tone audiometry or, in children, with brainstem-evoked response audiometry (BERA). Degree of hearing loss was computed by using a four-frequency average called the pure-tone average. The average of the hearing threshold levels (decibels) taken at 500 Hz, 1000 Hz, 2000 Hz, and 4000 Hz was calculated for each patient to find the pure-tone average level. Afterwards, hearing loss was graded according to the classification defined by J.G. Clark in 1981 [36]. Dystopia canthorum, which is characterized by an increase in the distance between the inner angles of the eyelids with normal distances between the pupils and the outer canthus, was defined for each affected member of the family with a W index that exceeds 1.95 [1].

### Mutation analysis

Peripheral blood samples which were collected in EDTA tubes and stored fresh (-20 °C) were obtained from participating members of the family and 50 unrelated healthy volunteers. Genetic analyses were performed in Duzen Laboratory Groups, Genetics Division, Ankara, Turkey. Genomic DNA was extracted from leukocytes using the QIAamp DNA mini kit (Qiagen, 51304, Dusseldorf, Germany), according to manufacturer's instructions. Genomic fragments including coding regions and adjacent intronic regions of *PAX3* were amplified with PCR, using nine primer pairs described previously [7] (Table 1). The amplicons from individual exons were purified and analyzed with cycle sequencing with ABI BigDye Terminator Cycle Sequencing Kit v3.1 (ABI Applied Biosystems, Foster City, CA) on an automatic DNA sequencer (ABI 3130 Genetic Analyzer, Applied Biosystems). Sequencing results from patients and the consensus sequences from the NCBI Human Genome Database (NCBI Reference Sequence: NG_011632.1) were imported into the ABI SeqScape program and aligned to identify variations. Each found mutation was confirmed with bidirectional sequencing. The mutation was named following the nomenclature recommended by the Human Genomic Variation Society.

**Table 1:** Primers for amplifying and sequencing *PAX3* genomic fragments [7].

Exon	Forward primers	Reverse primers	Product size (bp)
1	5′ GATGGGAAGAGAAAGTGGTC 3′	5′ TGCAGAAAGGAAATCGAGTA 3′	788
2	5′ CCGATGTCGAGCAGTTTCAG 3′	5′ CGCACCTTCACAAACCTCAG 3′	503
3	5′ TGGGATGTGTTCTGGTCTG 3′	5′ TCCCAATAGCTGAGATCGA 3′	420
4	5′ CTGGAGAAGGATGAGGATGT 3′	5′ CGTCAGATCACCAATGTCAG 3′	383
5	5′ TACGGATTGGTTAGACTTGT 3′	5′ AACAATATGCATCCCTAGTAA 3′	508
6	5′ CAACACAGAAGGCAGAGA 3′	5′ ATAGGTACGTTCAGGACAA 3′	445
7	5′ TGTGCAGAGATAGGTGTGAC 3′	5′ TTTGATGAAGCCAGTAGGA 3′	586
8	5′ TCTCCTGGACAGCTCTTTAA 3′	5′ GGCATGTGTGGCTTAATCT 3′	480
9&10	5′ GGTCAGCTCCAGGATCATAT 3′	5′ GCAAATGGAATGTTCTAGCT 3′	580

## Results

### Phenotype analysis

Eight affected patients (five women [III:7, IV:3, IV:4, IV:5, and IV:6] and three men [II:3, III:5, and III:9]) and five nonaffected members (three women [III:2, IV:1, and IV:2] and two men [II:1 and III:1]) of three generations in the study family were enrolled in the present study (Figure 1). Case I:1 was not evaluated in this study as he died before enrollment. The diagnostic criteria for WS proposed by the Waardenburg Consortium and the clinical features of the study patients are shown in Table 2.

**Table 2:** Diagnostic criteria of WS, and clinical evaluation of affected family members. Two major, or one major and two minor criteria have to be found in an individual to be diagnosed as WS

DIAGNOSTIC CRITERIA	FEATURES	STUDY PARTICIPANTS							
		II-3	III-5	III-7	III-9	IV-3	IV-4	IV-5	IV-6
MAJOR CRITERIA	Sensorineural hearing loss	-	+	-	+	-	-	+	+
	Iris pigmentary abnormality (heterochromia iridis, or segmentary heterochromia of the iris, or characteristic brilliant blue iris)	-	+	+	+	-	-	+	+
	Hair hypopigmentation (white forelock, white hairs at other sites on the body)	-	-	-	-	-	-	-	-
	Dystopia canthorum	+	+	+	+	+	+	+	+
	First-degree relative previously diagnosed with Waardenburg syndrome	+	+	+	+	+	+	+	+
MINOR CRITERIA	Skin hypopigmentation	-	-	-	+	-	-	-	-
	Synophrys	+	+	+	+	-	+	-	-
	Broad nasal root	-	-	-	-	+	-	+	+
	Hypoplasia alae nasi	+	-	+	-	-	-	-	+
	Premature graying of the hair (before the age of 30 years)	+	+	+	+	N/A	N/A	N/A	N/A

**N/A:** Not applicable

**Figure 2:**
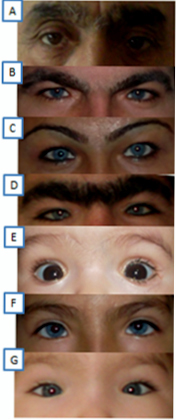
Photographs of eyes from patients with Waardenburg syndrome type 1. **A**: Dystopia canthorum (W index: 2.03) and synophyris were present in a 68-year old man (II:3). **B**: Dystopia canthorum (W index: 2.08), brilliant blue iris, and synophyris were present in a 32-year old man (III:5). **C**: Dystopia canthorum (W index: 1.96), brilliant blue iris, and synophyris were present in a 30-year old woman (III:7). **D**: Dystopia canthorum (W index: 2.35), brilliant blue iris, and synophyris were present in a 26-year old man (III:9). **E**: Dystopia canthorum (W index: 2.49) and broad nasal root were present in a 5-month old girl (IV:3). **F**: Dystopia canthorum (W index: 2.19), brilliant blue iris, and broad nasal root were present in a 10-year old girl (IV:5). **G**: Dystopia canthorum (W index: 2.39), brilliant blue iris, and broad nasal root. W index: 2.39.

**Figure 3:**
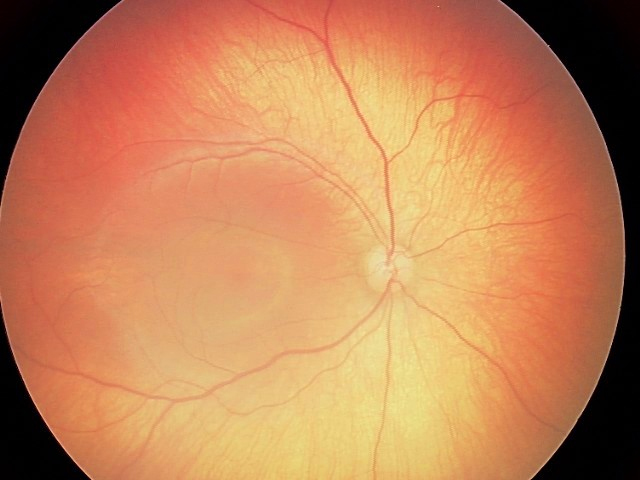
Retinal hypopigmentation was seen on the fundus photograph of the proband.

Dystopia canthorum was detected in all affected patients (Figure 2). A brilliant blue iris was present in five patients who also had mild retinal hypopigmentation (III:5, III:7, III:9, IV:5, and IV:6; Figure 2 and Figure 3). Orthoptic assessments were within normal limits, and nystagmus was not present in affected patients or nonaffected members of the family; however, astigmatic refractive errors were found frequently in all affected members. Synophrys was presented in five out of eight patients (II:3, III:5, III:7, III:9, and IV:4), and this finding was more prominent in two (II:3 and III:9; Figure 2). Clinical features of all unaffected family members (II:1, III:1, III:2, IV:1, and IV:2) were revealed as normal.

Four patients had a sensorineural hearing impairment. Three (III:5, IV:5, and IV:6) were profoundly and one (III:9) was moderately deaf. Out of the three profoundly deaf patients, two (IV:5 and IV:6) had received a cochlear implant. None had a white forelock, whereas premature graying of the hair (before the age of 30 years) was present in patients II:3, III:5, III:7, and III:9. Due to the patients’ youth, premature graying of the hair was not evaluated in four patients (IV:4 was 15 years old, IV:5 was 10 years old; IV:3 and IV:6 were younger than 2 years old). Hypopigmented patches of the skin were present in only one patient (III:9; Table 2). No patients had limb defects.

### Mutation analysis

**Figure 4:**
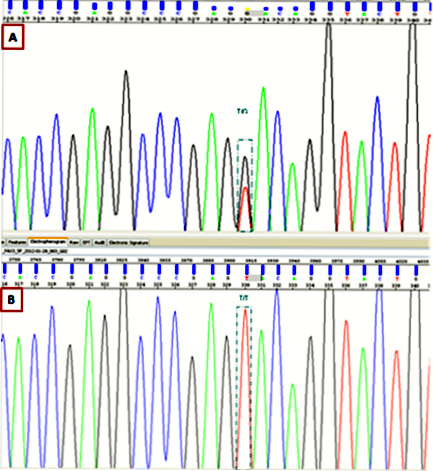
Sequence chromatography. **A**: The heterozygous change, c.788T>G, was identified in affected family members. **B**: Unaffected members and 50 healthy controls are wild-type at this position.

After direct sequencing of *PAX3*, a heterozygous change T>G in exon 5, at position 788 of the translation start site was detected in all affected patients (Figure 4A). This position belongs to helix 3 of the homeodomain in the PAX3 protein, and converts the 45^th^ amino acid in this domain from valine to glycine. This mutation was not found in the other unaffected relatives (II:1, III:2, IV:1, and IV:2) in the healthy controls (Figure 4B). Case III:1 declined the molecular genetic analysis.

## Discussion

A careful clinical evaluation is necessary to differentiate various types of WS. Dystopia canthorum is a commonly seen feature of WS with an incidence of 41.2% to 99% [1,2]. However, dystopia canthorum is also the most penetrant (%99) and the most distinguishing feature of WS1 [1,2,6,37,38]. All affected patients in the present study had dystopia canthorum.

In 2006, the frequencies of heterochromia iridis and synophrys were reported as 25% and 45% of WS1 patients, respectively [6]. However, incidence of synophrys in patients with WS1 was reported as 85% in a review published in 2009 [17]. Although a brilliant blue iris was present in five out of eight patients, heterochromia iridis was not detected in any members of the study family. However, synophrys, a minor criterion, was also present in five out of eight patients.

WS accounts for approximately 2% to 5% of congenital sensorineural deafness [30,39]. Congenital sensorineural deafness is a feature in approximately 25% to 75% of patients with WS1 [17,40,41]. Deafness related to WS can be moderate to severe, unilateral, or bilateral but most commonly non-progressive [40,42]. In our study, three patients had total bilateral severe sensorineural deafness, and one patient had moderate bilateral deafness. Hearing tests were normal in the other four affected patients and five unaffected members of the family. However, Wang et al. reported a lack of deafness among their Chinese patients with WS1, and indicated a possible ethnic specific variation in clinical expression of the syndrome [43]. The incidence of hearing loss among Turkish patients with WS1 was published as 75% by Oysu et al. in 2000 [40]. Hearing loss was present in 50% of our study patients with WS1, which is in accordance with the literature [17,30,39-42].

Mutations of *PAX3* on chromosome 2q37 have been reported in 33% to 80% of patients with WS1 in familial and sporadic cases [3,7,13,44]. *PAX3* is a transcription factor that plays a major role in embryogenesis [45]. By 2009, about 70 mutations of *PAX3* related to WS had been introduced [17]. The PAX3 protein is a member of the family of paired domain proteins that bind DNA and regulate gene expression [7]. *PAX3* encodes a paired domain and a homeodomain [6]. Missense mutations are almost exclusively located within the two DNA binding domains. One, the homeodomain, includes three α-helices. Most *PAX3* mutations are located in the paired domain or in helix 3 of the homeodomain [6]. Helix 3 of the homeodomain makes sequence-specific DNA contacts and several phosphate contacts in the major groove [6,7]. Mutations that affect helix 3 of the homeodomain in the PAX3 protein may lead to a decrease in DNA binding affinity and/or specificity [7]. We identified the novel c.788T>G mutation in *PAX3* leading to V→G substitution on the 45^th^ position of helix 3.

The phenotypic variability among the eight affected patients with the same mutation is well matched with inheritance properties of the disease. *PAX3* mutations are inherited dominantly with variable expressivity [43]. The disease is known as fully penetrant when at least one of its signs is detected, but the penetrance for each one is not complete. The fact that there is no obvious association between different types of mutations and the severity of disease could be due to the role of gene dosage as the pathophysiology of the syndrome. It is hypothesized that stochastic events were not solely responsible for its expression, so that genetic factors and/or the environmental background can modify the phenotype [17]. A few mutations of *PAX3* have been tested for their functional consequences. The functional experiments mostly included DNA-binding activity and transactivation capabilities. Although missense mutations are thought to abolish *PAX3* ability to bind and activate its transcriptional targets, further functional studies are necessary to evaluate the precise molecular mechanism caused by the c.788T>G mutation.

In conclusion, we identified a novel missense mutation in *PAX3* that is associated with the occurrence of WS1. The new mutation, like all other defined mutations, lead to phenotypic variability within the same family, which is one of the most important features of the disease.
